# Soluble CD146 reflects altered endothelial and metabolic homeostasis in peripheral artery disease

**DOI:** 10.1007/s10456-026-10064-6

**Published:** 2026-06-09

**Authors:** Haizam Oubari, Julien Labreuche, Charlotte Reytier, Françoise Dignat-George, Jean-Paul Duong-Van-Huyen, Joseph Emmerich, Pascale Gaussem, Patrick Bruneval, Marcel Blot-Chabaud, Alexandre G. Lellouch, Nathalie Bardin, David M. Smadja

**Affiliations:** 1 Center for Engineering in Medicine and Surgery, Department of Surgery, Harvard Medical School, Massachusetts General Hospital, États-Unis, Boston, MA États-Unis d’Amérique; 2 Service de Chirurgie Plastique, Reconstructrice Et Esthétique, Hôpital de La Croix-Rousse, Hospices Civils de Lyon, Lyon, France; 3 Department of Biostatistics, CHU Lille, 59000 Lille, France; 4 INSERM, INRAE, C2VN, Aix-Marseille Université, Marseille, France; 5 Service d’Hématologie Et de Biologie Vasculaire, CHU La Timone, AP-HM, Marseille, France; 6 Pathology Department, Necker Hospital, Assistance Publique-Hôpitaux de Paris, Paris, France; 7 Vascular Medicine Department, Saint Joseph Hospital, INSERM CRESS 1153, Paris Cité University, Paris, France; 8 AP-HP, PARCC, Hematology Department, European Georges Pompidou Hospital, 56 Rue Leblanc, 75015 Paris, France; 9 Paris Cité University, INSERM PARCC, Paris, France; 10 Cedars-Sinai Medical Center, Los Angeles, CA USA; 11 Service d’Immunologie Biologique, CHU La Timone, AP-HM, Marseille, France

**Keywords:** Peripheral arterial disease, Soluble CD146, HDL

## Abstract

**Background:**

Peripheral Arterial Disease (PAD) is a prevalent but underdiagnosed pathology. Soluble CD146 (sCD146) was described as a marker of endothelial dysfunction and vascular congestion.

**Objective:**

We hypothesize that sCD146 may represent a novel biomarker of PAD. Our objective was to evaluate the association between plasma sCD146 levels and the occurrence and severity of PAD.

**Methods:**

In this case–control study, 184 Caucasian men with symptomatic PAD were compared to 163 age-matched healthy control patients. PAD diagnosis was confirmed using ankle-brachial index (ABI) and imaging. Plasma sCD146 was quantified using ELISA. Associations with clinical and biochemical parameters were analyzed through multivariable logistic regression models.

**Results:**

sCD146 level was significantly reduced in PAD patients (mean [95% CI]: 288 ng/mL [269–306]) versus control patients (480 ng/mL [460–500], *p* < 0.0001). A 10 ng/mL decrease in sCD146 was associated with an age-adjusted odds ratio (OR) of 1.17 (95% CI 1.13–1.22) for PAD, increasing to OR 1.25 (95% CI 1.14–1.36, *p* < 0.0001) after adjustment for risk factors. sCD146 was not associated with PAD severity (by Fontaine stage). Notably, the association between sCD146 and HDL-C was positively correlated in controls (β = 0.220, *p* = 0.005), but negatively correlated in PAD patients (β = − 0.156, *p* = 0.041), with significant interaction (*p* = 0.002). Age-adjusted OR for PAD was highest in individuals with high HDL-C tertiles (OR = 1.41, 95% CI 1.22–1.63).

**Conclusion:**

A lower sCD146 concentration is independently associated with PAD. Additionally, an inverse relationship was observed between HDL-C and these patients. These findings suggest that sCD146 may reflect impaired endothelial homeostasis and metabolic dysregulation in PAD, indicating that it could serve as a diagnostic biomarker for the pathology.

*Clinical trials registration*: NCT00377897.

## Introduction

Peripheral artery disease (PAD) is a manifestation of systemic atherosclerosis that predominantly involves the arteries supplying the lower limbs and affects approximately 15% of individuals older than 55 years [1]. Clinically, PAD most commonly presents as either exertional leg discomfort or advanced ischemic complications. The typical early symptom, known as intermittent claudication, consists of exercise-induced muscle pain that reliably subsides with rest. In contrast, chronic limb-threatening ischemia (CLTI) represents the most advanced stage of the disease, occurring when arterial perfusion is insufficient to satisfy the metabolic requirements of tissues even at rest. This severe impairment may lead to persistent pain, non-healing wounds, tissue loss, and, in many cases, limb amputation. Despite its prevalence and serious consequences, PAD remains both under-recognized and inadequately managed. A substantial proportion of patients do not receive evidence-based therapies shown to lower cardiovascular risk and mortality, including antiplatelet medications, statins, and inhibitors of the renin–angiotensin–aldosterone system [1]. The ankle–brachial index (ABI) is currently the primary diagnostic tool used to detect PAD; however, it correlates poorly with disease severity. Beyond ABI, dependable diagnostic measures are limited, underscoring the need for novel biomarkers to improve assessment and risk stratification.

CD146 is a component of the endothelial junction and is involved in the control of paracellular permeability [2] and the regulation of monocyte transendothelial migration [3]. Besides its structural role, CD146 is also involved in cell signaling [4] and has been implicated in the angiogenic process [5, 6]. CD146 also exists as a soluble form (sCD146) which results from a metalloprotease-dependent shedding of membrane CD146 [3, 7]. sCD146 constitutes a vascular biomarker released during preeclampsia [8] but also in response to venous stretch and serves as an indicator of systemic congestion in heart failure [9, 10]. It is associated with imaging markers of volume overload on echocardiography and shows increased concentrations in patients with both preserved and reduced left ventricular ejection fraction. Notably, this elevation can be observed even in cases where circulating B-type natriuretic peptide (BNP) values remain within a low range [9]. Unlike BNP, sCD146 is not influenced by myocardial injury, making it useful in acute coronary syndrome and emergency settings. It constitutes an additive diagnostic biomarker when combined with N-terminal pro BNP (NT-proBNP), especially in patients with intermediate levels. In hemodialysis patients, sCD146 helps to distinguish between cardiac and non-cardiac causes of overhydration [11]. Overall, it is a promising tool for assessing congestion and guiding treatment in heart failure.

The aim of this study was to investigate whether sCD146 could serve as a biomarker for PAD. To this end, we measured sCD146 in 184 PAD patients and 163 controls in a case–control study [12–14].

## Methods

### Study cohort

Over a 24-month interval, 184 individuals with peripheral artery disease (PAD) were prospectively recruited from our vascular medicine unit. Eligibility criteria included Caucasian males younger than 70 years presenting with symptomatic lower-extremity atherosclerosis, defined by an ankle–brachial index (ABI) below 0.90 or a prior history of surgical or endovascular limb revascularization, in accordance with previously established criteria [12–14]. Patients were excluded if lower-limb ischemia was attributable to non-atherosclerotic etiologies such as cardioembolic events, thromboangiitis obliterans, inflammatory vasculitis, or congenital/metabolic vascular disorders. Chronic limb-threatening ischemia (CLTI) was diagnosed in the presence of rest pain or a persistent ulcer lasting longer than two weeks combined with an ankle systolic pressure < 50 mmHg. Claudication severity was assessed using the patient-reported maximal walking distance prior to symptom-induced cessation of ambulation. The control population comprised 163 men without a personal history of arterial cardiovascular disease (including stroke, myocardial infarction, angina, or PAD). These controls were randomly selected from a previously characterized cohort of 703 Caucasian men enrolled for investigation of genetic determinants of vascular thrombosis [12]. They had been referred to a specialized cardiovascular prevention center for routine health evaluation. All participants provided written informed consent. The study protocol received approval from the Paris-HEGP-Broussais institutional ethics committee. Detailed baseline characteristics of PAD cases have been described previously [14].

Disease severity was categorized according to the Fontaine–Leriche (WHO) classification:

- Stage I: intermittent claudication with walking distance > 100 m
- Stage II: intermittent claudication with walking distance < 100 m
- Stage III: chronic limb-threatening ischemia

Data regarding lipid-lowering and antiplatelet therapies are summarized in Table 1.

**Table 1:** Main patient characteristics of PAD cases and controls. Values are no./total no. (%), mean (standard deviation) or median (25th to 75th percentiles); for quantitative variables, in case of missing data, the number of available cases are reported in bracket [n =].

	PAD cases (N = 184)	Controls (N = 163)	Standardized Differences, %
Age, years	57.1 ± 7.2	53.5 ± 6.3	52.4
Body mass index, kg/m^2^	24.4 ± 4.1 [n = 182]	25.6 ± 3.1	− 32.3
Medical history			
Diabetes	45/178 (24.5)	11/162 (6.8)	52.1
Current smoking	108/184 (58.7)	43/163 (26.4)	69.2
Hypertension	97/184 (52.7)	37/163 (22.7)	65.1
Hyperlipidemia	111/184 (60.3)	86/163 (52.8)	15.3
Lipid-lowering treatment	80/180 (44.4)	12/163 (7.4)	93.4
Antiplatelet therapy	132/182 (72.5)	19/163 (11.7)	156.7
Biological data			
Fasting glucose, mmol/l	5.7 (5.2 to 6.7) [n = 177]	5.8 (5.5 to 6.2) [n = 162]	
Total cholesterol, mmol/l,	5.0 ± 1.2	5.9 ± 0.9	− 72.2
LDL-C, mmol/l	3.0 ± 1.0 [n = 164]	4.1 ± 1.0 [n = 158]	− 104.1
HDL-C, mmol/l	1.1 ± 0.3 [n = 171]	1.2 ± 0.6 [n = 160]	− 31.7
Triglycerides, mmol/l	1.5 (1.2–2.3)	1.2 (0.8–1.7)	64.4
TSP-1 (ng/mL)	478 (204) [n = 175]	251 (177) [n = 104]	118.3
VEGF-A (pg/mL)	16.4 (8.4 to 26.8)	13.3 (9.5 to 18.1) [n = 106]	24.8
PlGF (pg/mL)	8.2 (5.4 to 11.7)	7.3 (5.7 to 8.4) [n = 146]	29.8

HDL-C = high-density lipoprotein cholesterol; LDL-C = low-density lipoprotein cholesterol; PlGF** = **placental-derived growth factor; TSP-1 = Thrombospondin-1; VEGF-A = Vascular Endothelial Growth Factor-A

### Blood sampling and measurement of plasma sCD146

Venous blood was drawn into citrate-anticoagulated Vacutainer tubes (0.105 M sodium citrate; Becton–Dickinson Diagnostics, Le Pont-de-Claix, France) using a 1:9 anticoagulant-to-blood ratio. Samples were centrifuged at 2300 g for 10 min to obtain platelet-poor plasma, which was then aliquoted and stored at –80 °C pending analysis. Circulating soluble CD146 (sCD146) concentrations were quantified using a commercially available enzyme-linked immunosorbent assay (ELISA) kit (CY-QUANT ELISA, Biocytex®, Marseille, France), following the manufacturer’s instructions. This ELISA kit used to measure sCD146 in plasma has been thoroughly characterized and validated in the original development study [15]. This assay is based on two distinct monoclonal antibodies directed against CD146. Both antibodies are well-characterized and highly specific to CD146. The specificity of these antibodies has been established in the original validation study, which reported excellent coefficients of variation (repeatability: 8.2%; reproducibility lot-to-lot: 2.9%; within-batch reproducibility: 3.5%), and normal reference values were determined in a large cohort of 293 healthy donors.

### Intramuscular administration of bone marrow–derived mononuclear cells in critical limb ischemia

Patients with critical limb ischemia (CLI) were enrolled in the OPTIPEC study (Optimization of Progenitor Endothelial Cells in the Treatment of Critical Leg Ischemia; ClinicalTrials.gov identifier: NCT00377897), a multicenter, phase I, open-label trial. Clinical outcomes from this study have been reported previously [16, 17]. Inclusion criteria required the presence of CLI with limited gangrene or a non-healing ischemic ulcer in patients deemed unsuitable for conventional revascularization procedures (surgical bypass or percutaneous angioplasty), or in whom such interventions were unlikely to be effective. The protocol for bone marrow mononuclear cell (BM-MNC) implantation was adapted from the pioneering approach described by Tateishi-Yuyama et al. [18]. For histological comparison, tissue samples were obtained from age- and sex-matched patients with CLI who underwent limb amputation during the same timeframe but did not receive cell therapy.

### Statistical analysis

Continuous data are presented as mean ± standard deviation when normally distributed, and as median with interquartile range (25th–75th percentile) when distributional assumptions were not met. Categorical data are summarized as counts with corresponding percentages. Distribution normality was evaluated graphically using histograms and formally tested with the Shapiro–Wilk procedure. Main patient’s characteristics were described according to the two study groups, and the magnitude of the between-group differences was assessed by calculating the absolute standardized difference; an absolute standardized difference > 20% was interpreted as a meaningful difference [19]. We initially compared the sCD146 concentrations between PAD and control patients by using analysis of covariance (ANCOVA), adjusted for age, as well as among the PAD cases according to disease severity. Given the well-established imbalance in major cardiovascular risk factor (diabetes and smoking) between PAD patients and controls, comparisons were stratified according to these variables by including the corresponding interaction term into ANCOVA model. We further assessed the magnitude in between-group comparisons by calculating the area under the receiver operating characteristic (ROC) curve (AUC); from the ROC, we determined the low and high thresholds providing high sensivity and high specificity (≥ 90%) respectively. The shape of the association was further investigated in a logistic regression model using restricted cubic splines. Since there was no deviation from the log-linear assumption, the effect of sCD146 concentration was fitted using only a linear term and odds ratio (OR) with its 95% confidence interval (CIs) for PAD versus control per one 10 ng/ml decrease was estimated as the effect size. Association of sCD146 concentrations and PAD was further adjusted for age and pre-specified confounders (body mass index, medical history (diabetes, current smoking, hypertension, hyperlipidemia, lipid-lowering treatment, and antiplatelet therapy), fasting glucose, and lipid profiles (LDL-C, HDL-C, and triglycerides). We further investigated the association of sCD146 concentrations with main patients’ characteristics separately in PAD and control patients using separate linear regression models adjusted for age; standardized regression coefficients were reported as effect size. Since a negative association between sCD146 and HDL-C concentrations was found according to the case–control status, we tested all first-order interactions between patients’ characteristics and case–control status in an age-adjusted analysis of covariance model. To further describe the interaction with HDL-C levels, we estimated the age-adjusted ORs of PAD versus control per 10 ng/ml decrease in sCD146 levels according to HDL-C tertile levels. All hypothesis tests were two-sided, with a threshold for statistical significance set at *p* < 0.05. Analyses were performed using SAS software (version 9.4; SAS Institute, Cary, North Carolina, USA).

## Results

### Study population

Table 1 shows the main demographic and medical characteristics of the study population. A strong imbalance in patients’ characteristics was found, except for fasting glucose level. In comparison with the control group, individuals with PAD were approximately sixfold more frequently treated with lipid-lowering medications, which was reflected in reduced concentrations of total cholesterol and LDL cholesterol. Despite this, they exhibited a more atherogenic lipid profile overall, characterized by elevated triglyceride levels and decreased HDL cholesterol. A significant difference in the use of antiplatelet therapy was observed (72.5% in PAD vs. 11.7% in controls). We also found that PAD patients displayed a lower mean body mass index than control patients. Among the PAD patients, lower limb arterial angiography was available for 110 patients (60%), and all patients had an ultrasound examination of the aorta and lower limb arteries in the course of the disease, confirming the diagnosis of atherothrombotic PAD. Most cases had intermittent claudication with a median walking distance of 200 m. The ABI was 0.65 ± 0.13 in patients with claudication and 0.56 ± 0.24 in patients with claudication and/or chronic limb threatening ischemia.

### Plasma sCD146 concentrations are decreased in patients with peripheral arterial disease

**Fig. 1:**
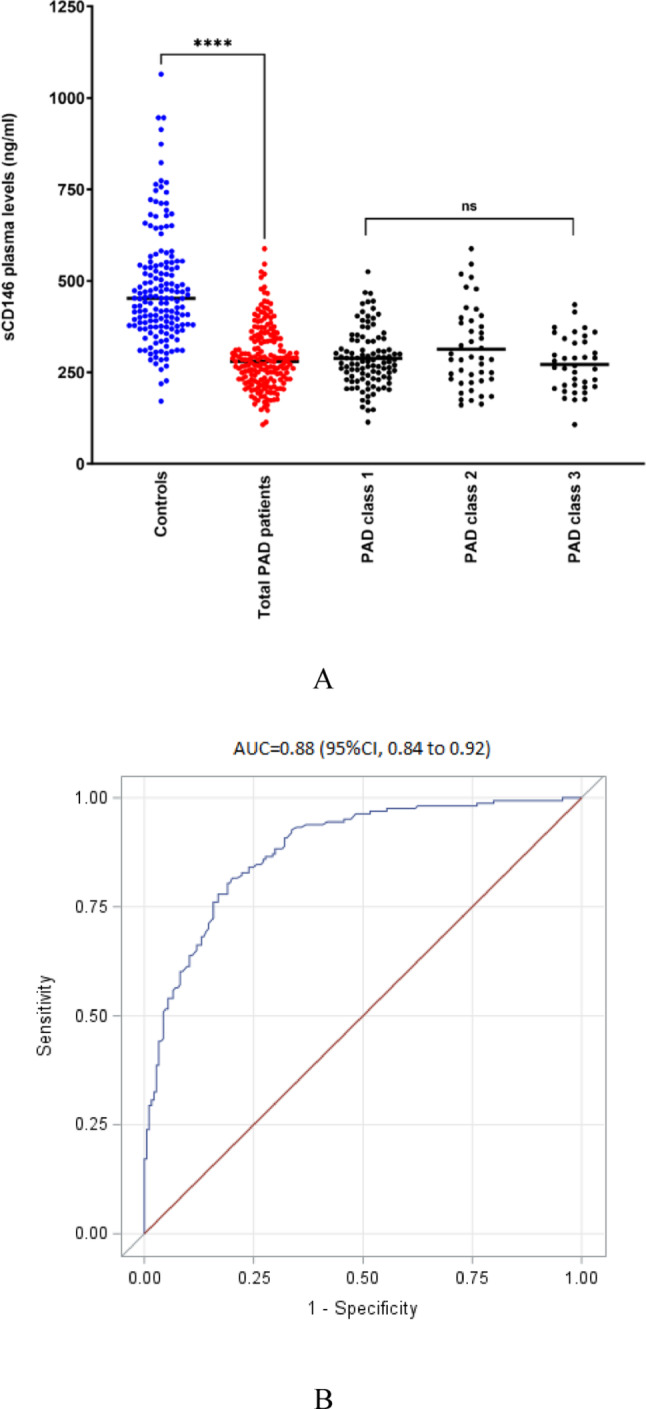
Plasma sCD146 levels are decreased in PAD and demonstrate strong diagnostic performance **A** Distribution of sCD146 plasma concentrations in patients with PAD and control individuals. **B** Receiver operating curve (ROC) for discriminating PAD versus control by sCD146 levels. The area under ROC (AUC) was 0.88 (95%CI 0.84 to 0.92), with a grey zone between 193 and 710, corresponding to thresholds ensuring 90% sensitivity and 90% specificity, respectively

**Fig. 2:**
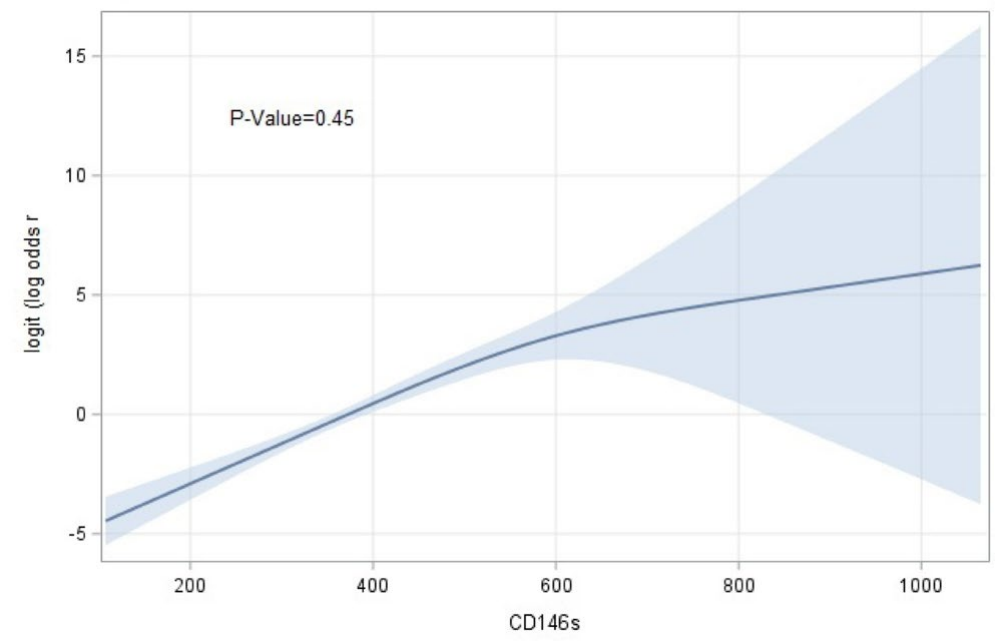
Shape of the association of CD146s levels with PAD status in logistic regression analysis adjusted for age. The fitted value of logit of PAD (PAD versus Controls) per increase in CD146s levels at the mean age (55 years, calculated in overall cohort) are reported. P -Values of the likelihood ratio test comparing the model including restricted cubic spline terms the model including a linear term only are reported

As shown in Fig. 1A, plasma sCD146 concentrations were significantly lower in PAD patients (age-adjusted mean 288 ng/ml [95%CI 269 to 306)]) than in control patients (480 ng/ml [460 to 500]). In analysis restricted to PAD patients, we found no significant difference in plasma concentrations of sCD146 according to disease severity (Fig. 1A). Given the well-established imbalance in major cardiovascular risk factors—particularly diabetes and smoking—between PAD patients and controls, we performed stratified analyses of sCD146 levels according to these variables. As shown in Table 2, sCD146 concentrations remained significantly lower in PAD patients compared with controls across all subgroups, including both diabetic and non-diabetic individuals, as well as current smokers and non-smokers. Furthermore, stratification by PAD severity revealed no significant differences in sCD146 levels across disease stages within these subgroups (Table 3). Collectively, these findings indicate that the association between reduced sCD146 levels and PAD is independent of diabetes and smoking status. In a receiver operating characteristic (ROC) analysis, sCD146 discriminates PAD patients from controls, with an area under the curve (AUC) of 0.88 (95% CI 0.84–0.92), indicating excellent discrimination performance (Fig. 1B). Clinically relevant thresholds were identified, with low sCD146 levels (< 193 ng/mL) achieving high sensitivity (approximately 90%) for the detection of PAD, and high levels (> 710 ng/mL) providing high specificity (approximately 90%) for ruling out the disease. These thresholds delineate an intermediate ‘grey zone’ between 193 and 710 ng/mL. In logistic regression model adjusted for age, sCD146 concentrations were linearly associated with the logit of PAD status (Fig. 2), with an age-adjusted OR for PAD versus control of 1.17 (95%CI 1.13 to 1.22) per 10 ng/ml decrease in sCD146 concentrations. Comparable effect sizes were observed after controlling for potential confounders, including age, major cardiovascular risk factors (hypertension, dyslipidemia, diabetes, and smoking), metabolic parameters such as glucose and lipid levels (as well as use of lipid-lowering therapy), and antiplatelet medication. The association remained statistically significant, with an adjusted odds ratio of 1.25 (95% CI 1.14–1.36; *p* < 0.0001).

**Table 2:** Comparison in sCD146 levels of PAD versus control according to subgroups defined by diabetes and smoking status. Values are age-adjusted means (95%CI) of sCD146 concentrations. P Het indicates p values for heterogeneity

	PAD cases (n = 184)	Controls (n = 163)	P	P het
*Diabetes*				
No	284 (264 to 304)	481 (461 to 500)	< 0.0001	0.44
Yes	302 (261 to 342)	447 (326 to 567)	0.026	
*Current smoking*				
No	280 (251 to 310)	482 (459 to 504)	< 0.0001	0.57
Yes	292 (269 to 316)	477 (439 to 515)	< 0.0001

**Table 3:** Comparison in sCD146 levels between PAD severity according to subgroups defined by diabetes and smoking status (subgroups defined by the two main established risk factors of PAD). Values are age-adjusted means (95%CI) of sCD146 concentrations. P Het indicates p values for heterogeneity

	PAD class 1 (n = 102)	PAD class 2 (n = 44)	PAD class 3 (n = 38)	P	P het
*Diabetes*					
No	282 (264 to 300)	303 (275 to 333)	278 (242 to 315)	0.41	0.086
Yes	346 (294 to 397)	272 (215 to 329)	295 (253 to 338)	0.14	
*Current smoking*					
No	289 (264 to 315)	302 (245 to 360)	270 (226 to 314)	0.63	0.67
Yes	288 (264 to 313)	296 (267 to 326)	297 (259 to 334)	0.90

### Plasma sCD146 concentrations are correlated with HDL-C concentrations

**Fig. 3:**
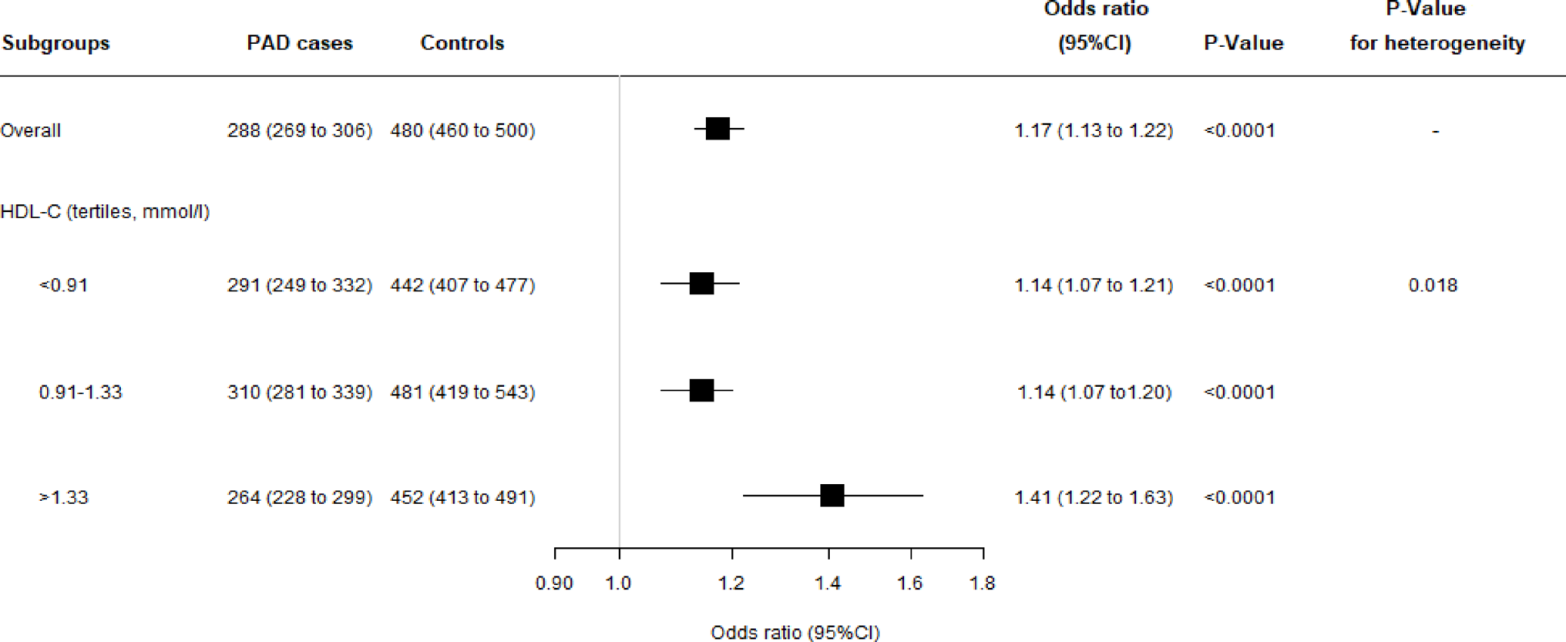
Age-adjusted odds ratios of PAD versus control per 10 ng/ml decrease in sCD146 levels, overall and according to HDL-C tertiles. Values are age-adjusted means (95%CI) of sCD146 concentrations unless otherwise as indicated

We then investigated whether circulating sCD146 levels were influenced by established PAD risk factors or by plasma mediators involved in angiogenesis, including pro-angiogenic factors (VEGF-A, PlGF) and the anti-angiogenic protein thrombospondin-1 (TSP-1). No significant relationships were observed between sCD146 concentrations and conventional clinical variables such as age, body mass index, diabetes status, active smoking, hypertension, dyslipidemia, or the use of statins and antiplatelet therapies. No association was found with VEGF-A, PlGF or TSP-1. However, we found that sCD146 concentrations were associated with HDL-C concentrations in both PAD and control patients (Table 4). Of interest, the association was found in two opposite directions in PAD and control patients. Indeed, the association between sCD146 and HDL-C concentrations was found to be positive in control patients and negative in PAD patients, indicating a statistically significant interaction between the two parameters. In the analysis of covariance adjusted for age, the interaction between case–control status and HDL-C concentrations was significant (*p* = 0.002), whereas all other interactions were not significant (*p* > 0.10). In the logistic regression model stratified by tertiles of HDL-C, the age-adjusted OR of PAD versus controls was significant across all HDL-C tertiles, with a stronger association for the highest tertile (age-adjusted OR per 10 ng/ml decrease in sCD146 levels of 1.41; 95%CI 1.22 to 1.63) (Fig. 3).

**Table 4:** Association of of sCD146 levels with mains patient’s characteristics according to PAD cases status. β indicates age-adjusted standardized regression coefficients (calculated using separate multiple linear regression model)

	PAD cases, n = 184	Controls, n = 163		
	β (95%CI)	*P-*Value	β (95%CI)	*P-*Value
Age	0.046	0.54	0.128	0.11
Body mass index	− 0.015	0.84	− 0.044	0.58
Diabetes	0.111	0.14	0.096	0.21
Current smoking	0.031	0.70	− 0.002	0.98
Hypertension	− 0.082	0.29	− 0.048	0.55
Hyperlipidemia	− 0.062	0.41	− 0.097	0.22
Lipid− lowering treatment	− 0.057	0.46	0.092	0.25
Antiplatelet treatment	− 0.021	0.78	− 0.079	0.32
Fasting glucose	0.11	0.16	0.001	0.99
Total cholesterol	− 0.049	0.51	0.053	0.50
HDL cholesterol	− 0.156	**0.041**	0.220	**0.005**
LDL cholesterol	− 0.024	0.77	− 0.048	0.55
Triglycerides	0.050	0.50	− 0.017	0.83
TSP− 1** (**ng/mL**)**	0.071	0.35	− 0.020	0.83
VEGF− A (pg/mL)	< 0.001	1.00	0.089	0.36
PlGF (pg/mL)	− 0.012	0.88	0.001	0.99

### CD146 is expressed by vessels in patients with peripheral arterial disease (PAD)

**Fig. 4:**
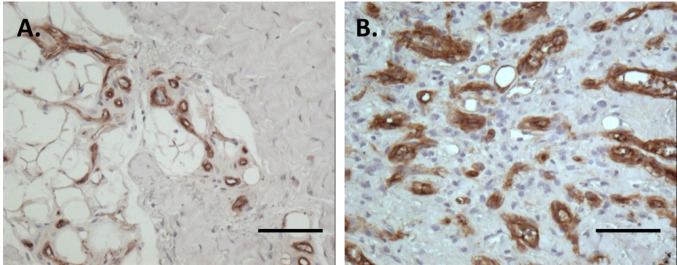
CD146 localization in neovessels following cell-based therapy in peripheral artery disease. Immunohistochemical analysis of amputation tissue from patients with critical limb ischemia demonstrates vascular CD146 staining in specimens obtained from untreated individuals (**A**) and from patients who received intramuscular bone marrow–derived mononuclear cell (BM-MNC) therapy (**B**). Scale bar: 50 µm

To confirm the relevance of studying sCD146 in PAD, we explored CD146 expression in distal tissues from amputated control patients with CLTI. We observed CD146 expression in all examined vessels in CLTI pathology samples (Fig. 4A). We also explored CD146 expression in specimens from patients treated with BM-MNCs (OPTIPEC trial) [16, 20]. All newly formed vessels examined expressed CD146, unlike other cell types (Fig. 4B). These results suggested that, at least in PAD patients, CD146 is a good endothelial surface marker.

## Discussion

In this case–control study, we showed for the first time a significant decrease in sCD146 concentrations in the plasmas of patients with PAD as compared to control patients. To our knowledge, this is the first description of an association between sCD146 concentration and PAD. Our findings, showing a significant decrease in plasma sCD146 concentrations in patients with PAD, contrast sharply with studies reporting increased sCD146 levels in chronic kidney disease (CKD), especially diabetic nephropathy (DN), and other pathologies related to vascular inflammation [21, 22]. In DN and in kidney transplant recipients, CD146 overexpression has been associated with glomerular endothelial injury, albuminuria, and adverse renal outcomes, suggesting that its shedding generates CD146, reflecting active endothelial stress and structural compromise [23, 24]. In our PAD cohort, however, lower sCD146 concentrations may signal a distinct endothelial phenotype; one characterized by endothelial exhaustion, reduced CD146 expression, or impaired proteolytic cleavage. These mechanisms are supported by findings in systemic sclerosis and neuroinflammatory diseases, where certain sCD146 variants are functionally profibrotic or pro-permeability, highlighting the complexity and heterogeneity of CD146-mediated vascular regulation [25, 26].

CD146 is a major component of endothelial intercellular junctions, and its shedding is a key event in endothelial activation, barrier destabilization, and the transmigration of immune cells [25, 27]. In vitro studies demonstrate that sCD146 promotes vascular permeability through integrin αvβ1 and participates in Wnt/β-catenin signaling, both of which are implicated in pathological angiogenesis and fibrosis [26]. A reduction in circulating sCD146 in PAD may therefore indicate defective junctional dynamics or a blunted endothelial regenerative response, consistent with the impaired angiogenesis observed in these patients [6, 28]. CD146 has been shown to enhance tumor angiogenesis [10], and the short form, in particular, enhances the angiogenic properties of endothelial progenitor cells both in vitro and in vivo [6]. sCD146 was also found to have angiogenic properties and promote neovascularization in experimental hind limb ischemia [29]. Consistent with prior work showing elevated TSP-1 and impaired angiogenesis in PAD [14], we also observed higher TSP-1 levels in PAD patients; however, we did not detect a direct association between TSP-1 and sCD146 concentrations in plasma in either PAD or control patients. This suggests that, within the limits of our cross-sectional sampling and analytic sensitivity, TSP-1 does not linearly track with sCD146 at the circulating level. Nonetheless, given TSP-1’s anti-angiogenic actions and its potential influence on endothelial junctional dynamics [30, 31], a context-dependent interaction, such as a tissue-restricted interaction between TSP-1 and CD146, remains biologically plausible. Future studies integrating paired tissue-plasma analyses, sCD146 isoform quantification, time-resolved sampling, or other mechanistic approaches are warranted to determine whether a physiological correlation exists between TSP-1 and CD146. Given that CD146 is a well-established endothelial marker expressed on vascular structures as demonstrated in Fig. 4, it may hold potential as a dynamic biomarker for disease monitoring in prospective studies. While our current cross-sectional analysis did not reveal differences in sCD146 levels according to PAD stage, longitudinal data at the individual level would be instrumental in determining the clinical relevance and temporal behavior of plasma sCD146 concentrations.

Interestingly, we observed a paradoxical reversal correlation in the relationship between sCD146 and HDL cholesterol in patients with peripheral artery disease (PAD) compared to control patients. While the association was positive in healthy individuals, it was negative in the context of PAD**.** This suggests a shift in the regulatory or compensatory roles of HDL and sCD146 in endothelial homeostasis under pathological conditions. Although direct mechanistic data remain sparse, CD146 may intersect with HDL metabolism through several pathways: endothelial lipid uptake regulation**,** scavenger receptor activity**,** or shared modulation by inflammatory cytokines [32]. Recent work by Zocchi et al. proposed that in obesity, a state marked by low HDL and chronic inflammation, HDL dysfunction and reduced adiponectin contribute synergistically to endothelial impairment, possibly reflecting a disrupted HDL-adiponectin-CD146 axis [33]. Moreover, CD146 is increasingly recognized as a vascular injury marker that reflects endothelial activation and angiogenic stress, and its levels correlate with adverse remodeling in atherosclerosis and diabetes [34]. Adiponectin, a protective adipokine with anti-inflammatory and pro-endothelial effects, has been shown to correlate positively with sCD146 in early vascular disease and kidney transplant cohorts [35, 36]. This association may reflect a compensatory response to subclinical endothelial stress. However, in PAD, where adiponectin and HDL-C are typically reduced, the disruption of this axis may signify a failure of vascular repair, leading to microvascular rarefaction, inflammation, and metabolic atherogenesis. Interestingly, adiponectin, a key adipokine with anti-inflammatory and vasculoprotective properties, has been shown to correlate positively with CD146 levels in several renal and transplant studies [23, 35]. Elevated adiponectin and CD146 may reflect a compensatory endothelial-protective axis in early vascular injury. However, in our PAD population, where adiponectin is known to be reduced [37], the loss of this axis may contribute to endothelial dysfunction and metabolic inflammation. Adiponectin is also a strong positive determinant of HDL-C and facilitates cholesterol efflux via ABCA1 expression, thus linking endothelial health, lipid metabolism, and inflammation [36]. Altogether, the inverse relationship between sCD146 and HDL concentrations in PAD may not only mirror altered lipoprotein-endothelial interactions but also underscore the breakdown of a protective signaling network involving adiponectin and HDL in atherogenic states. A key limitation of this study relates to treatment-related confounding. PAD patients were more frequently treated with lipid-lowering and antiplatelet agents, both of which could theoretically influence endothelial activation or lipid metabolism. These treatments were recorded and included in our multivariable models, and neither was significantly associated with sCD146 concentrations. Nonetheless, because this is an observational case–control design, residual confounding by treatment intensity, duration, or other concomitant medications not captured in our dataset cannot be completely excluded.

Our data point to a complex interplay among sCD146, HDL, and adiponectin, reflecting convergent endothelial injury, impaired junctional integrity, and dysregulated metabolic signaling. The inverse correlation between sCD146 and HDL in PAD, combined with the known reduction in adiponectin, suggests that these markers may function as integrated reporters of vascular stress and dysfunction, with potential diagnostic relevance. These observations also raise the hypothesis that CD146-related pathways might modulate vascular repair responses in PAD; however, further studies will be necessary to further validate sCD146 as a clinical biomarker in PAD.

## Data Availability

No datasets were generated or analysed during the current study.
